# Multigene phylogeny, morphology, and pathogenicity uncover two novel *Globisporangium* species (Oomycota) from freshwater habitats in northwestern Iran

**DOI:** 10.3389/fmicb.2025.1615096

**Published:** 2025-08-06

**Authors:** Reza Ahadi, Alireza Alizadeh, Ali Chenari Bouket, Hossein Masigol, Hans-Peter Grossart

**Affiliations:** ^1^Department of Plant Protection, Faculty of Agriculture, Azarbaijan Shahid Madani University, Tabriz, Iran; ^2^East Azarbaijan Agricultural and Natural Resources Research and Education Centre, Plant Protection Research Department, Agricultural Research, Education and Extension Organization (AREEO), Tabriz, Iran; ^3^Plankton and Microbial Ecology, Leibniz Institute for Freshwater Ecology and Inland Fisheries (IGB), Berlin, Germany; ^4^Bermuda Institute of Ocean Sciences, St. George's, Bermuda; ^5^Institute of Biochemistry and Biology, University of Potsdam, Potsdam, Germany

**Keywords:** aquatic oomycetes, plant parasites, *Pythium sensu lato*, systematics, taxonomy

## Abstract

During the study of oomycete biodiversity in aquatic environments of northwestern Iran (East Azarbaijan), four *Globisporangium* isolates were recovered from a river and irrigation canal. These isolates were identified based on multi-locus phylogenetic analyses (ITS, *cox1*, and *cox2* genomic regions) and morphological features. As a result, two novel species were described, namely *Globisporangium parvizense* sp. nov. and *G. sarabense* sp. nov., both exhibiting unique sporangial structures and growth patterns. Pathogenicity assays on cucumber seedlings confirmed strains' high potential to cause root and crown rot. This research highlights the diversity of *Globisporangium* in Iranian freshwater habitats, providing insights into its taxonomy and phylogenetic relationships. Detailed morphological descriptions and illustrations are provided for these novel species.

## 1 Introduction

*Pythium* Pringsheim, nom. cons., sensu lato (s.l.) is a diverse and ecologically important genus within the phylum Oomycota (commonly known as water molds), comprising over 200 species (Uzuhashi et al., 2010; Derevnina et al., 2016). As a member of the kingdom Straminipila which also includes diatoms, brown algae, and slime molds (Mueller, 2011; Watkinson et al., 2015), *Pythium* s.l. shares phylogenetic affinities with other important plant pathogenic genera such as *Albugo* Roussel, *Aphanomyces* de Bary, *Bremia* Regel, *Peronospora* Corda, *Phytophthora* de Bary, *Plasmopara* Schroet., and *Saprolegnia* Nees. This genus is particularly notable for its wide distribution across both terrestrial and aquatic habitats and for its significant impact on agriculture, natural ecosystems, and even animal health (Robideau et al., 2011; Shreves et al., 2024; Masigol et al., 2025).

*Pythium* s.l. causes economic losses in vegetables, fruits, and ornamentals, resulting in damping-off, root and crown rot (Martin and Loper, 1999; Uzuhashi et al., 2010; Nzungize et al., 2012; Nguyen et al., 2022). Thriving in soil and aquatic agroecosystems, members of *Pythium* s.l. are frequently encountered in agricultural fields, nurseries, and greenhouses, where they persist in soil, water, plant debris, and even snow (Al-Sheikh and Abdelzaher, 2012; Chenari Bouket et al., 2015). Although capable of surviving in both aquatic and dryland environments (Barton, 1958; Abdelzaher and Kageyama, 2020), their motile zoospores, equipped with flagella, favor water for dispersal, which enhances their infectivity in moist conditions. Some species have even adapted to saline environments, further expanding their ecological range (Al-Sheikh and Abdelzaher, 2012).

Additionally, *Pythium* s.l. could be a potential threat to aquatic ecosystems, particularly when introduced into water by human activities (Abdelzaher and Kageyama, 2020). In fact, certain water-dwelling species such as *Pythium insidiosum*, could be responsible for severe infections in humans and dogs, causing “pythiosis,” a disease that affects cutaneous and vascular tissues (Hamlin et al., 2024). The ability of *Pythium* s.l. to infect multiple hosts across different ecosystems highlights its adaptability and pathogenic potential.

Given the ecological significance of *Pythium*, delineating its species boundaries is essential, as it lays the foundation for subsequent ecological and functional studies. In recent years, the taxonomy of *Pythium* s.l. has undergone significant transformations, primarily driven by advancements in molecular techniques (Villa et al., 2006). Molecular phylogenetic analyses revealed the paraphyletic nature of *Pythium* s.l., leading to proposals for its division into several distinct lineages. Initially, Lévesque and De Cock (2004) classified *Pythium* s.l. into 11 clades (A–K) based on phylogenetic data derived from the nuclear rDNA internal transcribed spacer region (ITS1–5.8S–ITS2) and the D1–D3 domains of the 28S rDNA. These clades were later reassessed through multigene phylogenetic analyses, which resulted in a revised classification that consolidated the original clades into 10 groups, with clade K reassigned to the newly established genus *Phytopythium* (Villa et al., 2006; Bala et al., 2010). Furthermore, Uzuhashi et al. (2015) proposed a major revision, restructuring *Pythium* s.l. into five distinct genera: *Pythium* sensu stricto (clades A–D), *Globisporangium* (clades E–G, I, and J), *Elongisporangium* (clade H), *Ovatisporangium* (clade K = *Phytopythium*), and *Pilasporangium*, which stands apart from the previously defined clades.

Several studies have focused on the isolation and identification of *Pythium* s.l. associated with aquatic ecosystems. In one of the earliest studies, Abdelzaher et al. (1995) reported *Pythium* group F from aquatic environments. Later, *P. myriotylum, P. ultimum* var. *sporangiferum* (Kageyama, 2010), and *P. rishiriense* (Rahman et al., 2015) were reported from water, soil and floating water from Rishiri Island, Japan. More novel *Pythium* species have been recently reported from different aquatic ecosystems in Japan (Uzuhashi et al., 2015; Abdelzaher and Kageyama, 2020), South Korea (Nam and Choi, 2019), and China (Chen and Zheng, 2019).

Although oomycetes have been studied in Iran for decades, the focus has traditionally been on plant pathogenic species in *Peronosporomycetes* (e.g., *Pythium*, Mostowfizadeh-Ghalamfarsa, 2015; Salmaninezhad and Mostowfizadeh-Ghalamfarsa, 2017, 2019, 2022, 2023; Salmaninezhad et al., 2022). Only recently, *Saprolegniomycetes* (Ghiasi et al., 2010; Masigol et al., 2018, 2019, 2020, 2022, 2023, 2025; Mirmazloomi et al., 2022) and, to a lesser extent, *Peronosporomycetes* (Ahadi et al., 2024) have been investigated taxonomically and ecologically in aquatic ecosystems. However, as research on aquatic oomycetes is still limited and geographically specific, this study aims to identify *Globisporangium* isolates from agroecosystems in East Azarbaijan province, Iran, using a combination of morphological analysis and multigene phylogeny, based on the nuclear rDNA ITS1-5.8S-ITS2 region and partial cytochrome C oxidase subunit I (*cox1*) and subunit II (*cox2*) sequences. Additionally, the potential pathogenicity of these isolates on cucumber, a common host plant of oomycetes, was evaluated.

## 2 Materials and methods

### 2.1 Sample collection and isolation

Sampling was conducted in the aquatic environments of East Azerbaijan Province, Iran (Table 1). Algae and roots of the grass species *Cynodon dactylon* were collected from a river and irrigation canal in 50 mL Falcon tubes and stored at 4°C prior to processing in the laboratory. Following surface sterilization for 1 min, tissue samples were cultured on NARF medium (Morita and Tojo, 2007) and incubated at 15°C for 5 days. Upon hyphal growth, a portion of the culture was transferred to WA medium (20.0 g/L agar) for purification using the hyphal tip method (Goh, 1999). Purified isolates were preserved on CMA medium in McCarthy vials at 10°C.

**Table 1:** Detailed information of isolates obtained in this study.

**Isolate**	**IRAN culture collection accession number**	**Matrix**	**Ecosystem**	**Location**	**Longitude**	**Latitude**
AZFC-RA178-2-1	IRAN 5217C	Root of *Cynodon dactylon*	Irrigation water	East A., Tabriz	37°9′54.8″	46°0′37.9″
AZFC-RA178-2-2	IRAN 5344C	Root of *Cynodon dactylon*	Irrigation water	East A., Tabriz	37°9′54.8″	46°0′37.9″
AZFC-RA98-1-1	IRAN 5173C	Algae	River	East A., Sarab	37°6′24.6″	47°6′15.9″
AZFC-RA98-1-2	IRAN 5345C	Algae	River	East A., Sarab	37°6′24.6″	47°6′15.9″

### 2.2 Morphological analysis

Growth patterns of the isolates were observed 2 weeks after inoculation on various agar media, including corn meal agar (CMA) (MIRMEDIA, Iran), potato dextrose agar (PDA) (Sigma Aldrich, Germany), malt extract agar (MEA) (DIFCO, USA), potato carrot agar (PCA) (Sigma Aldrich, Germany), and V8-juice agar (SIGMA, Germany) at 25°C. Morphological evaluations were performed on sexual and asexual structures produced on autoclaved hemp seeds and ryegrass pieces floating in sterile water from different sources (pond water, distilled water, tap water) (Martin, 1992). Twenty measurements were taken for each structure. Microscopic structures were photographed using a Nikon Eclipse Ti2 microscope with a digital camera system (Nikon, Japan). All purified cultures were deposited in the fungal culture collection of Azarbaijan Shahid Madani University, Tabriz, Iran (AZFC) and Iranian Research Institute of Plant Protection, Tehran, Iran (IRAN). Type specimens were also deposited in the herbarium of the Iranian Research Institute of Plant Protection.

The critical temperatures for growth were determined by incubating the strains on potato carrot agar (PCA) at 0, 2, 5, 10, 15, 20, 25, 30, 35, and 40°C. Descriptions were provided based on ex-type strains, and additional data for strains showing distinct morphological differences were included.

### 2.3 Phylogenetic analyses

Genomic DNA was extracted from 5-day-old CMA cultures using a modified manual procedure (Möller et al., 1992). Three genomic regions were targeted for amplification: the ITS-rDNA region, which contains the 5.8S nuclear ribosomal RNA gene and the internal transcribed spacers ITS1 and ITS2, and partial sequences of the mitochondrial *cox1* and *cox2* genes. These regions were amplified using specific primer pairs: ITS5 (GGAAGTAAAAGTCGTAACAAGG) and ITS4 (TCCTCCGCTTATTGATATGC) (White et al., 1990) for the ITS-rDNA region, FM55 (GGCATACCAGCTAAACCTAA) and FM52R (TTAGAATGGAATTAGCACAAC) (Martin, 2000) for the *cox1* gene, and FM58 (CCACAAATTTCACTACATTGA) and FM66 (TAGGATTTCAAGATCCTGC) (Villa et al., 2006) for the *cox2* gene. All reactions were conducted in a total volume of 50 μL, consisting of 25 μL of ready-to-use PCR Master mix (SinaClon, Iran), 1.2 μM of each primer, 18.6 μL of DNase-free water, and 10 ng of DNA. Amplifications were carried out using a PeqStar 96X universal thermal cycler with the following conditions: 95°C for 5 min, followed by 30 cycles of denaturation at 95°C for 30 s, annealing at 55°C for 30 s, and extension at 72°C for 1 min, with a final extension step at 72°C for 7 min for ITS-rDNA (White et al., 1990), and 94°C for 5 min, followed by 40 cycles of denaturation at 94°C for 30 s, annealing at 54°C for 30 s, and extension at 72°C for 1 min, with a final extension step at 72°C for 7 min for *cox1* and *cox2* genes (Martin, 2000; Chenari Bouket et al., 2015).

PCR amplicons were sequenced by Macrogen (Amsterdam, the Netherlands). Raw sequences were manually edited using SeqManII^®^ (DNA STAR) and MEGA v. 6 (Tamura et al., 2013). Sequence data from ex-type and reference strains of known *Globisporangium* species were obtained from NCBI GenBank (www.ncbi.nlm.nih.gov/genbank/) (Table 2). The retrieved sequences were assembled using Geneious (version 5.6) and aligned using the Q-INS-I algorithm in MAFFT (latest version), available on the MAFFT web server (Katoh and Standley, 2013; Katoh et al., 2019), separately for each of the genomic regions. Subsequently, after the removal of gaps, phylogenetic analyses were performed on the TrEase webserver (Mishra et al., 2023) for the individual genes using FastTree2 (Price et al., 2010) for Minimum Evolution, RAxML (Stamatakis, 2014) for Maximum Likelihood, and MrBayes (Ronquist et al., 2012) for Bayesian inference. For the Bayesian analysis, a GTR model was selected, and the analyses were run on random trees for 1,000,000 generations, discarding 30% of the first trees as burn-in steps of the analysis to determine posterior probabilities from the remaining trees. RAxML and FastTree2 trees were generated using the GTRGAMMA and GTR algorithms, respectively, and the reliability of the inferred trees was assessed through bootstrap analysis with 1,000 replications. After ensuring that there were no conflicting topologies in the phylogeny of the individual loci, they were concatenated, with the borders marked to ensure independent modeling of substitution rates for each partition. Multigene phylogenies (ITS, *cox1*, and *cox2*) with support values were calculated in the same manner as mentioned above, using three different approaches to assess the robustness of the inferred phylogenies. The sequences obtained in this study were deposited in GenBank, and their accession numbers have been provided in Table 2.

**Table 2:** A list of strains studied, with collection details and GenBank accession numbers.

**Species name**	**Sample code**	**Locality**	**GenBank accession no**.		
			**ITS**	***cox*** **1**	***cox*** **2**
*G.abappressorium* ^T^	CBS110198	USA	HQ643408	HQ708455	KJ595409
*G. acanthophoron*	CBS33729	USA	HQ643413	HQ708460	KJ595376
*G.acrogynum* ^A/T^	CBS54988	China	HQ643414	HQ708461	AB362324
*G.alternatum* ^T^	CBS139279	–	AB998876	AB998877	–
*G.apiculatum* ^PN^	CBS120945	France	HQ643443	HQ708490	KJ595422
*G. attrantheridium*	DAOM230383	Canada	HQ643477	HQ708524	AB512886
*G.baisense* ^T^	QBS123	–	FR775440	FR774198	–
*G.barbulae* ^T^	CBS139569	Japan	LC028389	LC028392	LC028395
*G. breve*	HMAS 242231	–	FR751317	FR774196	–
*G. buismaniae*	CBS28831	Netherlands	HQ643479	HQ708526	KJ595368
*G.camurandrum* ^PN^	DAOM BR876	–	GQ244426	GQ244425	**–**
*G.canariense* ^T^	CBS112353	Spain	HQ643482	HQ708528	JX397983
*G. capense*	CBS 149752	Australia	OL342598	OL331986	OL332028
*G. carolinianum*	CBS122659	India	HQ643484	HQ708530	KJ595427
*G.cederbergense* ^T^	CBS133716	South Africa	JQ412768	JQ412793	JQ412805
*G. commune*	CBS 149753	Australia	OL952618	OL860920	OL860929
*G.coniferarum* ^T^	CBS148568	Iran	ON554847	MZ020754	MZ020759
*G. cryptoirregulare*	CBS118731	USA	HQ643515	HQ708561	GU071763
*G.cylindrosporum* ^T^	CBS21894	Germany	HQ643516	HQ708562	GU071762
*G. cystogenes*	CBS67585	Netherlands	AY707985	HQ708564	KJ595396
*G. debaryanum*	CBS75296	UK	HQ643519	HQ708565	KJ595399
*G.echinulatum* ^PN^	CBS28164	Australia	HQ643531	HQ708577	AB362327
*G. emineosum*	DAOM BR836	–	GQ244428	GQ244424	–
*G.erinaceum* ^PN^	CBS50580	New Zealand	HQ643534	HQ708578	AB362326
*G. glomeratum*	CBS122644	France	HQ643542	HQ708586	KJ595424
*G.heterothallicum* ^T^	CBS45067	Canada	HQ643553	HQ708597	AB512919
*G. hypogynum*	CBS23494	France	HQ643565	HQ708609	AB362325
*G. intermedium*	CBS26638	Netherlands	HQ643572	HQ708616	AB507410
*G.iranense* ^T^	IRAN2386C	Iran	MG182709	MG182705	–
*G. irregulare*	CBS250.28	–	GQ410356	GU071822	GU071760
*G. iwayamai*	CBS15664	Australia	HQ643669	HQ708713	JX397979
*G.izadpanahii* ^T^	CBS144006	Iran	MK454537	OP321103	MK455859
*G. jasmonium*	DAOM229150	USA	HQ643670	HQ708714	–
*G. kandovanense*	CBS 139567	Iran	KP723167	KP938427	KP723171
*G.kunmingense* ^T^	CBS55088	China	HQ643672	HQ708716	KJ595389
*G. lacustre*	MAFF 236903	Japan	LC209786	LC209787	–
*G.longandrum* ^PN^	CBS112355	France	HQ643679	HQ708723	KJ595413
*G.longisporangium* ^PN^	CBS122646	France	HQ643680	HQ708724	KJ595426
*G. lucens*	CBS113342	Canada	HQ643681	HQ708725	KJ595415
*G. macrosporum*	CBS57480	Netherlands	HQ643684	HQ708728	AB512916
*G.mahabadense* ^T^	IRAN 4986C	Iran	PQ037626	PQ031213	PQ031206
*G. mahabadense*	IRAN 5253C	Iran	PQ037627	PQ031212	PQ031205
*G. mamillatum*	CBS25128	Netherlands	HQ643687	HQ708731	AB512918
*G. marsipium*	CBS77381	Netherlands	HQ643690	HQ708734	KJ595401
*G. mastophorum*	CBS37572	UK	HQ643691	HQ708735	KJ595378
*G. megalacanthum*	DAOM229154	Germany	HQ643693	HQ708737	KJ595435
*G.middletonii* ^PN^	CBS52874	Netherlands	HQ643694	HQ708738	AB362318
*G.minor* ^T^	CBS22688	UK	HQ643696	HQ708740	AB362320
*G.multisporum* ^T^	CBS47050	–	HQ643700	HQ708744	AB362319
*G. nagaii*	CBS77996	UK	HQ643705	HQ708749	KJ595402
*G. nodosum* ^T^	CBS102274	France	HQ643709	HQ708753	KJ595407
*G. nunn* ^T^	CBS80896	USA	HQ643711	HQ708755	–
*G. okanoganense* ^T^	CBS31581	USA	HQ643714	HQ708758	KJ595373
*G.ornacarpum* ^T^	CBS112350	France	HQ643721	HQ708762	KJ595411
*G. orthogonon*	CBS37672	Lebanon	HQ643723	HQ708764	KJ595379
*G. paddicum*	CBS69883	Japan	HQ643728	JX397975	JX397982
*G. papilogynum*	CBS122648	India	HQ643729	HQ708770	–
*G.paroecandrum* ^PN^	CBS15764	Australia	HQ643731	HQ708772	DQ071391
*G.parvum* ^T^	CBS22588	UK	HQ643738	HQ708779	AB362322
***G.parvizense* ^T^**	IRAN 5217C	Iran	PV444307	PV454623	PV454627
***G. parvizense***	IRAN 5344C	Iran	PV444308	PV454624	PV454628
*G. pleroticum*	CBS77681	Netherlands	HQ643748	HQ708789	AB362321
*G.polare* ^T^	CBS118203	Norway	KJ716859	–	KJ595417
*G. polymastum*	CBS81170	Netherlands	HQ643752	HQ708793	KJ595403
*G. radiosum*	CBS21794	France	HQ643756	HQ708797	KJ595356
*G. recalcitrans* ^T^	CBS 122440	Spain	DQ357833	EF426549	KJ595423
*G.rhizosaccharum* ^T^	CBS112356	India	HQ643760	HQ708801	AB362323
*G. rooibos*	STE-U7549	–	JQ412770	JQ412795	JQ412807
*G.rostratifingens* ^PN^	CBS115464	USA	HQ643761	HQ708802	KJ595416
*G.rostratum* ^PN^	CBS53374	Netherlands	HQ643767	HQ708808	KJ595388
***G.sarabense* ^T^**	IRAN 5173C	Iran	PV444306	PV454622	PV454626
***G. sarabense***	IRAN 5345C	Iran	PV444305	PV454621	PV454625
*G.segnitium* ^PN^	CBS112354	Spain	HQ643772	HQ708813	KJ595412
*G. selbyi*	CBS129729	USA	JF836871	JF895536	JF895532
*G. solare* ^T^	CBS119359	Spain	KJ716860	–	KJ595421
*G.spiculum* ^T^	CBS122645	France	HQ643790	HQ708831	KJ595425
*G. spinosum*	CBS27667	Netherlands	HQ643792	HQ708833	KJ595366
*G. splendens*	CBS46248	USA	HQ643795	HQ708836	AB512921
*G.sylvaticum* ^T^	CBS45367	USA	HQ643845	HQ708886	–
*G.tabrizense* ^T^	IRAN 4985C	Iran	PQ037624	PQ031210	PQ031204
*G. tabrizense*	IRAN 5254C	Iran	PQ037625	PQ031211	PQ031203
*G.takayamanum* ^T^	CBS122491	Japan	HQ643854	HQ708895	–
*G.tenuihyphum* ^T^	Chen 268	China	MF984123	MF984160	–
*G. terrestris*	UM2097	Australia	OL342605	OL331993	OL332034
*G. ultimum* var. *sporangiiferum*^T^	CBS21965	USA	HQ643879	HQ708920	AF196641
*G. uncinulatum*	CBS51877	Netherlands	HQ643944	HQ708985	KJ595385
*G. urmianum*	IRAN2376C	Iran	KT894049	KT894057	–
*G. viniferum*	CBS119168	Canada	HQ643956	HQ708997	KJ595419
*G. violae*	CBS15964	Australia	HQ643958	HQ708999	JX397980
*G.yorkense* ^T^	C12-118	USA	KY990050	KT692789	KY985298
*Phytopythium littorale*	CBS118360	Germany	HQ643386	HQ708433	KJ595418

-: Accession number not available. The specimen examined and the sequences generated in this study are indicated in bold.

### 2.4 Pathogenicity assays

Pathogenicity tests were conducted using a single isolate of each species (IRAN 5217C and IRAN 5173C, respectively). Cucumber (*Cucumis sativus* L.), a known host for a wide range of oomycetes (Ben-Yephet and Nelson, 1999; Lévesque and De Cock, 2004; Roberts et al., 2016; Sigillo et al., 2020; Zhang et al., 2023), was selected as the test plant. The inoculum was prepared according to the methods of Ahadi et al. (2024) with minor modifications. A sterile substrate containing sandy loam soil, wheat seed, and distilled water was inoculated with five mycelial plugs (5 mm^3^) from three-day-old PDA cultures of the target isolates. After 9 days of incubation at 25°C, cucumber seeds were sown and grown in a greenhouse at 25 ± 2°C with a 16-h photoperiod for 14 days. Positive controls consisted of plants grown in soil inoculated with each of the test isolates, while negative controls were grown in non-inoculated soil. Symptoms including wilting, crown and root rot, and stem discoloration and deterioration were assessed daily for 14 days. Symptomatic plants were further analyzed to isolate and identify potential pathogens using previously described isolation methods. A randomized complete block design with six replicates per treatment was employed.

## 3 Results

### 3.1 Phylogeny

In the analysis of multi-locus alignment (*Globisporangium* species), which included the gene boundaries of ITS (1–1,066), *cox1* (1,067–1,560), and *cox2* (1,561–2,069), a total of 89 isolates belonging to *Globisporangium*, along with an outgroup, were examined. The combined dataset (ITS + *cox1* + *cox2*) comprised 2,069 characters, including alignment gaps, with 958 variable characters (636 for ITS-rDNA, 169 for *cox1*, and 153 for *cox2*) and 1,002 constant characters (the sequence lengths were 333 bp for ITS-rDNA, 320 bp for *cox1*, and 349 bp for *cox2*).

**Figure 1:**
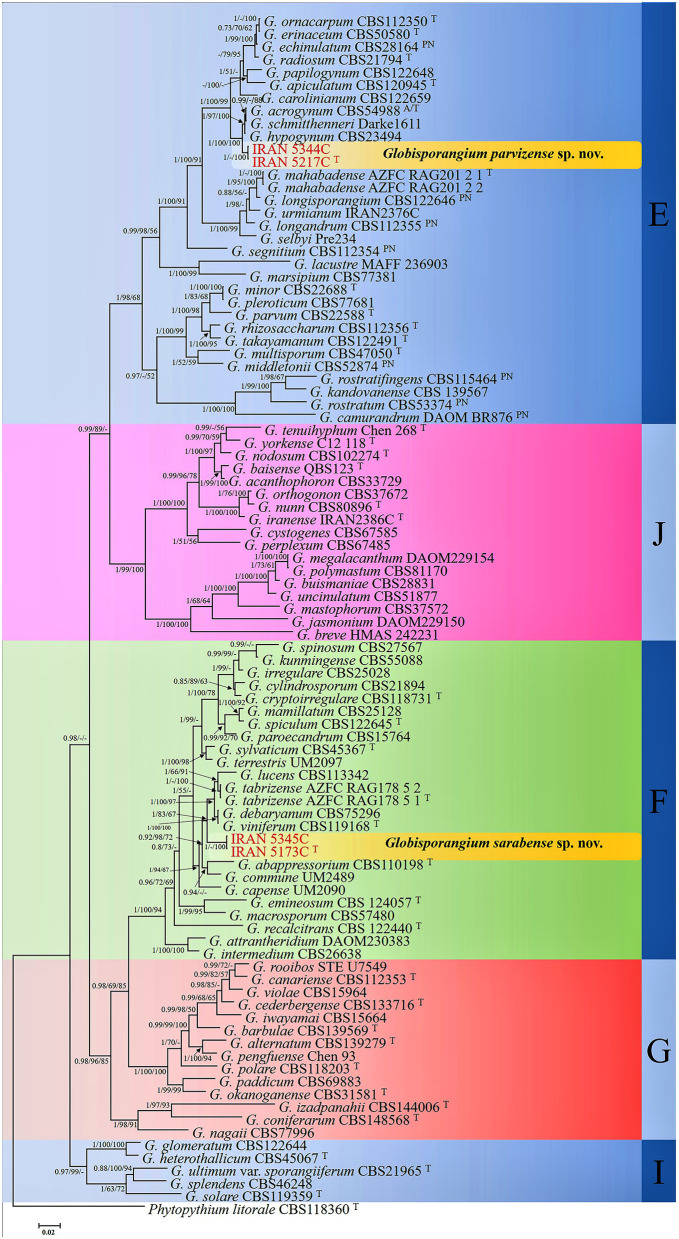
Phylogram generated from Bayesian inference analysis based on ITS-rDNA, *cox1*, and *cox2* sequence data for four examined strains and 89 reference strains of *Globisporangium*. Numbers on the branches represent posterior probabilities from Bayesian Inference, as well as bootstrap support from Minimum Evolution (ME) and Maximum Likelihood (ML), in the order 0.7/50%/50%. A dash indicates lower support for the presented topology or the possibility of an alternative topology. *Phytopythium litorale* type strain CBS118360 was used as the outgroup. Clades E–G, I, and J, which were identified by Lévesque and De Cock (2004) within the *Pythium sensu lato*, are depicted on the right side of the figure. Strains obtained in this study are shown in red. “T” denotes ex-type strains, and “PN” indicates authentic strains used for description in the monograph by Van der Plaats-Niterink (1981).

Bayesian analysis confirmed the tree topology obtained from minimum evolution (ME) and maximum likelihood (ML) methods. While most Bayesian posterior probability values were consistent with bootstrap supports, the Bayesian tree was selected to represent the phylogeny, with bootstrap values from ME and ML methods incorporated for comparison (Figure 1). In all three-locus phylogenetic trees (Bayesian, ML, and ME), the four *Globisporangium* isolates were placed into two distinct, well-supported clades, each representing a separate species. Specifically, the isolates IRAN 5217C and IRAN 5344C formed a robust monophyletic group, with maximum support from Bayesian posterior probability (1) and bootstrap values (100% for both ME and ML). Similarly, isolates IRAN 5173C and IRAN 5345C were clustered together in a separate, strongly supported clade [support values: 1.00 (Bayesian posterior probability), 83% (ME), and 67% (ML)]. In the multi-locus analysis, the topologies of the single-locus phylogenies did not conflict with the respective three-locus phylogenies, confirming the reliability and accuracy of the inferred relationships among these isolates.

### 3.2 Taxonomy

This study identified two *Globisporangium* species from various aquatic environments in Iran. Four isolates, including two from the roots of the grass species *Cynodon dactylon* growing in an irrigation canal (IRAN 5217C and IRAN 5344C), and two from water surface algae in the Sarab River in Sarab City (IRAN 5173C and IRAN 5345C) were reported as new species: *G. parvizense* sp. nov. and *G. sarabense* sp. nov.

**Figure 2:**
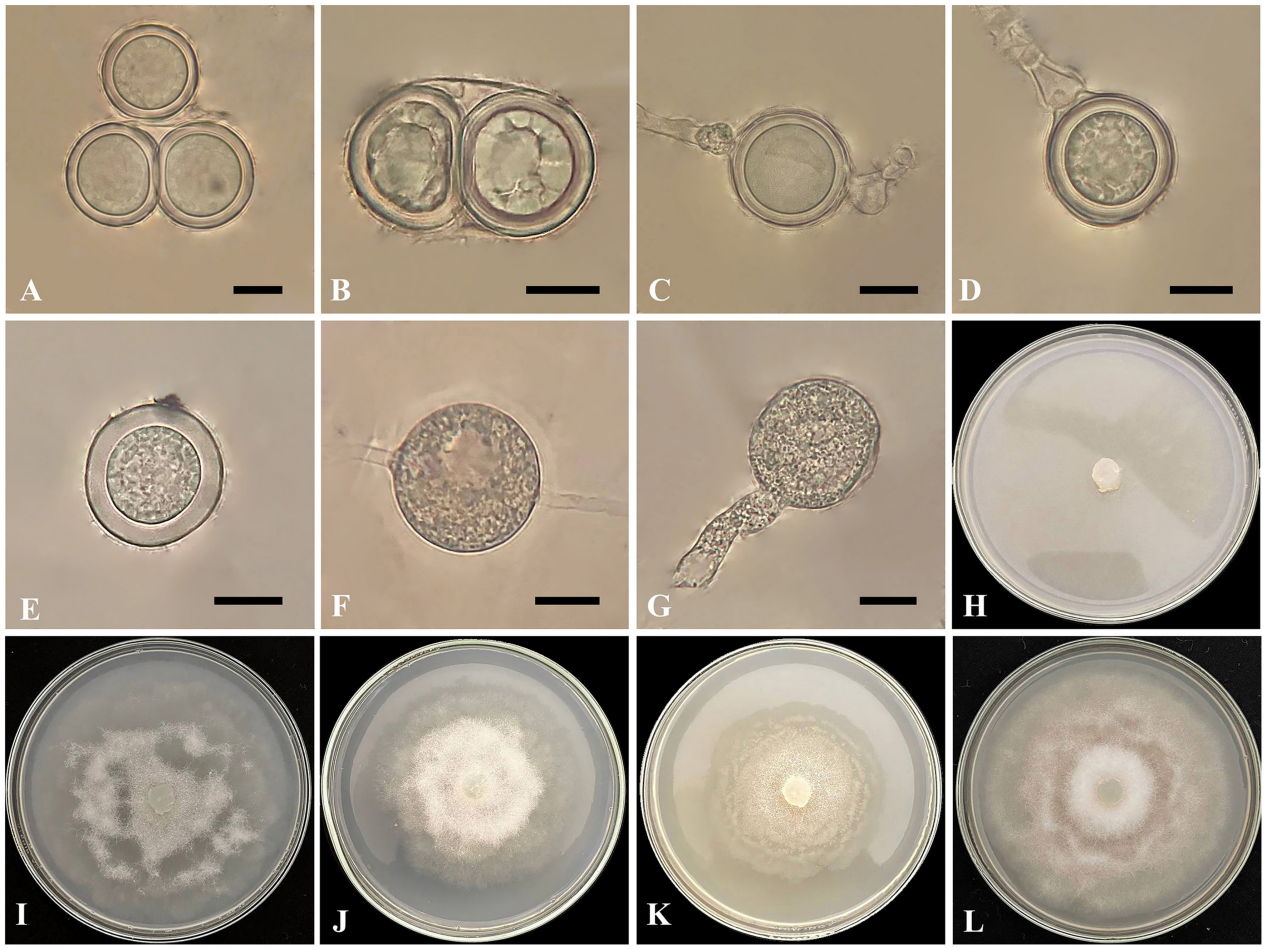
*Globisporangium parvizense* isolate IRAN 5217C. **(A, B)** Two oospores per oogonium. **(C)** Intercalary oogonium with one antheridium. **(D)** Terminal oogonium with one antheridium. **(E)** Plerotic oospore. **(F)** Intercalary sporangium. **(G)** Sporangium with discharge tubes. **(H)** Corn Meal Agar (CMA). **(I)** Potato Carrot Agar (PCA). **(J)** Potato Dextrose Agar (PDA). **(K)** Malt Extract Agar (MEA). **(L)** V8 Juice Agar (V8A). Scale bars: 10 μm.

*Globisporangium parvizense* Ahadi, Alizadeh, Chenari Bouket sp. nov. (Figure 2).

MycoBank: 857765

Typification: IRAN. East Azarbaijan: Tabriz (Varanaq), from roots of the grass species *Cynodon dactylon*, growing within the agricultural irrigation canals, Oct 2022, *R. Ahadi* (holotype IRAN 18632F). Ex-holotype culture IRAN 5217C = AZFC-RA178-2-1. GenBank: ITS = PV444307; *cox1* = PV454623; *cox2* = PV454627.

Etymology: The species is named in honor of the first author's father (Parviz), whose assistance was invaluable in the collection of the species.

Morphology: The colony pattern on MEA and PCA is a semi-rosette pattern, pattern on CMA is entirely within the growth medium and pattern on PDA and V8A appears submerged to stellate, with a daily growth rate of 9 mm at 25°C on PCA. The cardinal temperatures are at a minimum of 5°C, an optimum of 25°C, and a maximum of 30°C on PCA. The main hyphae are hyaline, aseptate, and range from 2.4 to 6 μm in width. The globose sporangia appear intercalary or terminal, measuring 11–32 μm in diameter (mean 23 μm). Globose hyphal swelling, terminal or intercalary, 8.5–16.5 μm (mean 10 μm) in diameter. Discharge tube arising from sporangia, short to long, one and rarely two per sporangia, and thick, 13–41 μm (mean 19.5 μm) in length and 7–8.5 μm (mean 8 μm) in width. Zoospores are not observed. Oogonia are globose and smooth, appearing intercalary or terminal, with a diameter range of 14–23 μm (mean 20.5 μm) and produced in single cultures. Antheridia are typically one, occasionally two per oogonium, diclinous. Oospores are plerotic, with one, rarely two per oogonium, measuring 15–23 μm in diameter (mean 20 μm). The wall thickness ranged from 1.8 to 3.6 μm.

Additional specimen examined: IRAN. East Azarbaijan: Tabriz (Varanaq), from roots of the grass species *Cynodon dactylon*, growing within the agricultural irrigation canals, Oct 2022, *R. Ahadi*. culture IRAN 5344C = AZFC-RA178-2-2. GenBank: ITS = PV444308; *cox1* = PV454624; *cox2* = PV454628.

Notes: Phylogenetic analysis revealed a close relationship between *Globisporangium parvizense* and *G. hypogynum*, followed by a slightly less close relationship with *G. acrogynum* and *G. schmitthenneri*. These species, as well as all other *Globisporangium* species, can be differentiated from *G. parvizense* based on ITS, *cox1*, and *cox2* sequence data. *Blastn* searches on NCBI GenBank revealed that the ITS sequence of the *G. parvizense* ex-type strain (IRAN 5217C = AZFC-RA178-2-1) exhibited 99% identity with the *G. hypogynum* type strain CBS23494 (HQ643565), *G. schmitthenneri* type strain Darke1611 (JF836869), and *G. acrogynum* isolate CBS54988 (HQ643414) (with two, three, and three nucleotide differences, respectively). Similarly, the *cox1* sequence of the ex-type strain shared 99% identity, respectively (with four nucleotide differences for both), with the *G. hypogynum* type strain CBS23494 (HQ708609), *G. acrogynum* isolate CBS54988 (HQ708461), and *G. schmitthenneri* type strain Darke1611 (JF895534). Comparative analysis of *G. parvizense* with the *cox2* sequence showed 98% identity, with seven nucleotide differences compared to *G. hypogynum* type strain CBS23494 (AB362325), *G. acrogynum* isolate CBS54988 (AB362324), and *G. schmitthenneri* type strain Darke1611 (JF895530). The phylogenetic data robustly establish *G. parvizense* as a phylogenetically isolated species within the genus *Globisporangium*.

*Globisporangium parvizense* is morphologically distinct from its phylogenetically close species based on several morphological characteristics. A detailed comparison of *G. parvizense, G. hypogynum, G. acrogynum*, and *G. schmitthenneri* reveals several distinct features. *G. parvizense* is characterized by narrower hyphae, up to 6 μm in width, compared to 7.7, 8, and 8.3 μm in *G. acrogynum, G. schmitthenneri*, and *G. hypogynum*, respectively. While *G. parvizense* exclusively forms globose sporangia, *G. schmitthenneri* also produces lemon-shaped sporangia. *Globisporangium parvizense* also has smaller oogonia (up to 23 μm) compared to *G. hypogynum* (up to 35 μm), and consistently smooth oogonia, unlike *G. acrogynum*. *Globisporangium parvizense* typically has one, occasionally two, antheridia per oogonium, while *G. schmitthenneri* can have up to three, and *G. acrogynum* has only one antheridium per oogonium. *Globisporangium parvizense* is uniquely characterized by the consistent formation of two spherical oospores per oogonium, while *G. acrogynum* and *G. hypogynum* each produce a single oospore. These morphological characteristics, including narrower hyphae, simpler sporangial shapes, consistent oospore formation, and unique antheridial structures, clearly differentiate *G. parvizense* from its closely related species (Table 3).

**Table 3:** Morphological comparison of *Globisporangium parvizense* sp. nov. and related species.

**Characteristics**	***G. parvizense* sp. nov**.	***G.schmitthenneri* ^a^**	***G.acrogynum* ^b^**	***G.hypogynum* ^b^ ^,^ ^c^**
Daily growth on PCA	9 mm	13.2 mm	Not reported	11 mm
Width of main hyphae	2.4–6 μm	4–8 μm	7.7 μm	1.5–8.3 μm
Discharge tube	Short to long, one and rarely two per sporangia and thick, 13–41 μm (av. 19.5 μm) length and 7–8.5 μm (av. 8 μm) width	Arising from the sporangia was long, >two times longer than sporangia and narrow	–	About twice the diameter of the sporangia
Zoospores	Not observed	7–10 mm long	Not observed	Observed
Sporangium	Globose, intercalary or terminal, 11–32 μm (av. 23 μm).	Terminal, occasionally intercalary, (sub) globose, lemon shaped, 17–43 (av. 32) μm, up to 48 μm long when limoniform	24–40 (av. 31) μm, terminal, rarely intercalary, (sub) globose	Terminal, occasionally intercalary, (sub) globose, non-proliferating, 6.5–34.5 (av. 22) μm
Oogonia	Globose, smooth, intercalary or terminal, 14–23 μm (av. 20.5)	Mostly terminal, occasionally intercalary, smooth 19–26 (av. 23) μm, oogonial stalk 3–10 μm	Terminal, (sub) globose, papillate, rarely smooth, 18–25 (av. 21) μm in diameter	Terminal, (sub) globose, smooth, 10–35 (av. 22) μm in diameter
Antheridia	One occasionally two per oogonium, diclinous	Mostly diclinous occasionally monoclinous and hypogynous, antheridial cells 10–25 × 6–12 μm, one occasionally as many as three per oogonia	One per oogonia, hypogynous, antheridial cells large, 8–15 × 6–14 (av. 11.5–9) μm	Strictly hypogynous, antheridial cells 3.0–11.1 × 2.8–8.3 (av. 6.5 × 5.5) μm delimited within the oogonial stalk at disitance of 5–30 μm below the oogonium
Oospore	Plerotic, one rarely two per oogonium, 15–23 μm (av. 20 μm)	Plerotic, single, occasionally two oospores per oogonium, 14–23 (av. 20) μm in diameter, wall smooth	Plerotic, single, 18–23 (av. 20) μm in diameter, wall smooth	Plerotic, single, smooth-walled
Wall thickness of oospore	1.8–3.6 μm	1–2.5 μm	0.8–1.7 (av. 1.5) μm	Not reported

a Description taken from Ellis et al. (2012).

b Description taken from Middleton (1941).

c Description taken from Van der Plaats-Niterink (1981).

**Figure 3:**
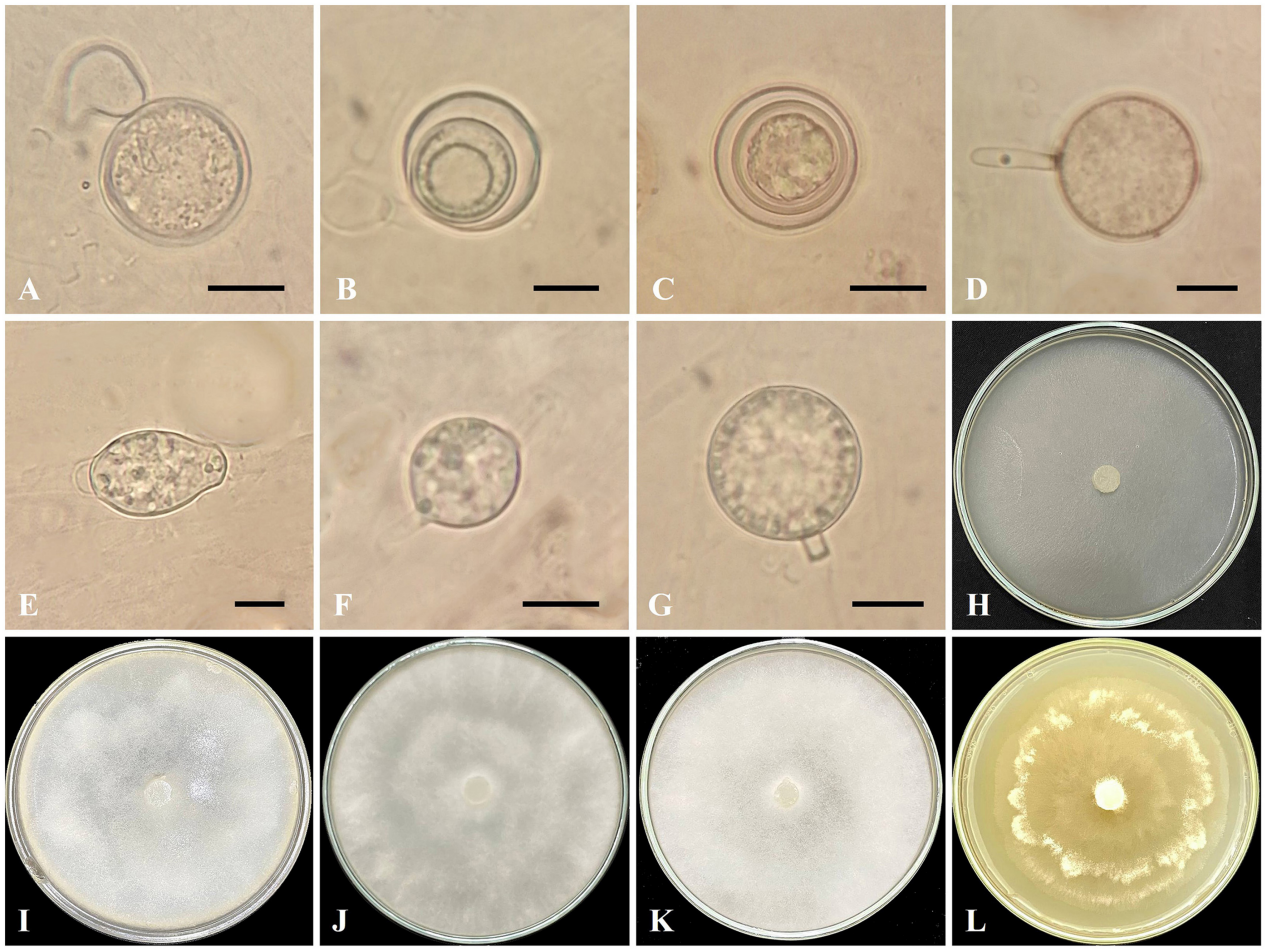
*Globisporangium sarabense* isolate IRAN 5173C. **(A)** Oogonium with one antheridium. **(B, C)** Aplerotic oospores. **(D)** Sporangium with discharge tubes. **(E, F)** Intercalary sporangium. **(G)** Terminal sporangium. **(H)** Corn Meal Agar (CMA). **(I)** Potato Carrot Agar (PCA). **(J)** Potato Dextrose Agar (PDA). **(K)** V8 Juice Agar (V8A). **(L)** Malt Extract Agar (MEA). Scale bars: 10 μm.

*Globisporangium sarabense* Ahadi, Alizadeh, Chenari Bouket sp. nov. (Figure 3).

MycoBank: 857791

Typification: IRAN. East Azarbaijan: Sarab, from water surface algae in Sarab River, Oct 2022, *R. Ahadi* (holotype IRAN 18544F). Ex-holotype culture IRAN 5173C = AZFC-RA98-1-1. GenBank: ITS = PV444306; *cox1* = PV454622; *cox2* = PV454626.

Etymology: Referring to the type location, Sarab City.

Morphology: The colony pattern on MEA is a rosette pattern, whereas the pattern on CMA, PDA, PCA, and V8A are submerged to stellate. Daily growth at 25°C on PCA is 20 mm. Cardinal temperatures are a minimum of 5°C, an optimum of 25°C, and a maximum of 35°C on Potato Carrot Agar. Main hyphae are hyaline, aseptate, and 2.4–6 μm (mean 3.1 μm) wide. Sporangia are globose or subglobose, intercalary or terminal, 17–26.8 μm (mean 21.6 μm) in diameter, and lemon-shaped sporangia are intercalary or terminal, 23.1–31.7 μm (mean 27.4 μm) in length and 17–26.8 μm (mean 21.7 μm) in width. The discharge tube is 12.8–10.3 μm (mean 11.5 μm) in length and 3–3.6 μm (mean 3.3 μm) in width. Zoospores are not observed. Oogonia are rarely formed, mostly terminal, smooth, globose, 18.3–23.1 μm (mean 20 μm) in diameter, and produced in a single culture. Antheridia are one per oogonium, mostly diclinous. Oospores are globose, aplerotic, one per oogonium, measuring 12.2–23.3 μm (mean 15.8 μm) in diameter, with wall thickness ranging from 0.6 to 2.4 μm.

Additional specimen examined: IRAN. East Azarbaijan, Sarab, from water surface algae in Sarab River, Oct 2022, *R. Ahadi*. culture AZFC-RA98-1-2. GenBank: ITS = PV444305; *cox1* = PV454621; *cox2* = PV454625.

Notes: Phylogenetic analysis revealed a close phylogenetic relationship between *G. sarabense, G. lucens*, and *G. tabrizense*, followed by a slightly less close relationship with *G. viniferum* and *G. debaryanum*. The ex-type strain of *G. sarabense* (IRAN 5173C = AZFC-RA98-1-1) exhibited 94% ITS identity with the *G. lucens* strain CBS113342, 96% identity in the *cox1* gene, and <96% identity in the *cox2* gene, as well as <94% ITS identity with the *G. tabrizense* strain IRAN 18502F. It showed 96.62% identity in the *cox1* gene and <97% identity in the *cox2* gene. However, it also displayed 21 and 15 nucleotide differences in ITS, 18 and 17 in *cox1*, and 10 and 12 in *cox2*, respectively, compared to this reference strain. Blastn searches on NCBI GenBank indicated that the ITS sequence of *G. sarabense* shared the highest similarity (97% identity, with 18 nucleotide differences and 5 gaps) with the *G. viniferum* isolate Tr-Sm01 (MT251151). Its *cox1* sequence was most similar (97%) to *Pythium* sp. isolate Kb003 (ON202818), and the *cox2* sequence was most similar (98%) to *G. intermedium* (MG256947). The ex-type strain of *G. sarabense* shared 96% identity in ITS, <95% in *cox1* and *cox2* genes with the *G. viniferum* voucher CBS119168, differing by 20 nucleotides in ITS, 16 in *cox1*, and 12 in *cox2*. Additionally, it shared 97% ITS identity, 96% *cox1* identity, and 97% *cox2* identity with *G. debaryanum* CBS75296, but exhibited 19 nucleotide differences in ITS, 16 in *cox1*, and 12 in *cox2*. The examined loci consistently placed *G. sarabense* in a separate phylogenetic clade, confirming its status as a distinct species within *Globisporangium*.

*Globisporangium sarabense* shares some morphological similarities with *G. lucens, G. tabrizense, G. viniferum*, and *G. debaryanum*. However, distinct morphological features differentiate *G. sarabense* from its congeners. Compared to *G. lucens, G. sarabense* lacks zoospores, while *G. lucens* produces zoospores. Furthermore, *G. sarabense* has single oospores, whereas *G. lucens* occasionally has two oospores per oogonium. Oogonia chains were not identified in *G. sarabense*, but *G. lucens* exhibits the formation of oogonia chains. In terms of sporangium morphology, *G. sarabense* exhibits greater diversity, including lemon-shaped, globose, or subglobose forms, while *G. lucens* primarily has globose or subglobose sporangia. *Globisporangium sarabense* possesses a single antheridium per oogonium, whereas *G. lucens* may have as many as five antheridia.

In contrast to *G. tabrizense, G. sarabense* is characterized by the absence of filamentous sporangia and the possession of a discharge tube, which further distinguishes it from *G. tabrizense*. Furthermore, in *G. sarabense*, the formation of oogonia is infrequent, and in contrast to *G. tabrizense*, the development of oogonia chains does not occur. In *G. tabrizense*, as many as two antheridia are produced, whereas in *G. sarabense*, only a single antheridium is formed. Oospores in *G. tabrizense* produce both plerotic and aplerotic types, with two oospores per oogonium. In contrast, *G. sarabense* only produces a single aplerotic oospore. Moreover, *G. viniferum* has up to three plerotic oospores per oogonium, but *G. sarabense* has a single, aplerotic oospore.

Differentiating *G. sarabense* from *G. debaryanum* is primarily based on the absence of zoospores and the production of only aplerotic oospores in *G. sarabense*. At the same time, *G. debaryanum* produces mostly plerotic and rarely aplerotic oospores. Additionally, in *G. sarabense*, the wall thickness of the oospore is 1.5 μm wider than in *G. debaryanum*.

In summary, *G. sarabense* is morphologically distinct from its congeners due to its unique combination of sporangia types, absence of zoospores, oospore characteristics, and number of antheridia. These morphological differences highlight the taxonomic distinctiveness of *G. sarabense* within the genus *Globisporangium* (Table 4 and Figure 3).

**Table 4:** Morphological comparison of *Globisporangium sarabense* sp. nov. and related species.

**Characteristics**	***G. sarabense* sp. nov**.	***G.tabrizense* ^a^**	***G.lucens* ^b^ ^,^ ^c^**	***G.viniferum* ^d^**	***G.debaryanum* ^e^**
Daily growth on PCA	20 mm	21 mm	9 mm	25 mm	30 mm
Width of main hyphae	2.4–6 μm	2.5–8.5 μm in diameter	3.5–6.5 μm in diameter	>7 μm in diameter	–
Sporangium	Globose, subglobose and lemon shape, terminal or intercalary, 17–31 (av. 24) μm; discharge tube single occasionally 2, 12.8–10.3 μm (mean 11.5 μm) length and 3–3.6 μm (av. 3.3 μm) width	Terminal and intercalary, lemon-shaped, filamentous, globose, 8.5–39.5 μm in diameter	Globose or subglobose, terminal and occasionally catenulate 2–3 in a chain, intercalary (21–25 μm in diameter); discharge tube up to 30 μm in diameter long, 1–2 per sporangium; Encysted zoospores 8–10 μm in diameter	Terminal and intercalary, spherical (7–25 μm in diameter), ovoid to elongated; at different temperatures, in sterile distilled, pond, and soil extract water	Spherical and terminal, up to 21 μm in diameter
Zoospores	Not observed	Not observed	8–10 μm in diameter	Zoospores were never observed in spite of repeated culturing	Present
Oogonia	Rarely formed, mostly terminal, smooth, globose, 18.3–23.1 μm	Intercalary and terminal, globose, smooth, 13.5–22 μm in diameter, rarely 2 in chain	Smooth walled, globose, 22–35 μm in diameter, rarely pyriform, usually terminal, occasionally intercalary, rarely 2–3 in chain	Mostly intercalary, occasionally terminal, sometimes catenulate, 17–29 μm in diameter, antheridia and oogonia borne on an appressorium, cylindrical or peanut-shaped	Spherical, smooth, 13–22 μm in diameter, terminal
Antheridia	One per oogonium, mostly diclinous	Diclinous and monoclinous, 1–2 per oogonium	1–2(5) per oogonium, monoclinous, usually stalked, originating usually more than 20 μm distance from the oogonium base, occasionally diclinous, antheridial cells clavate, occasionally 2–3 borne on one antheridial branch	Hypogynous, monoclinous sessile or monoclinous on short branches, at times diclinous, 1–5 per oogonium, antheridial cells conspicuous and at times bi-lobed, monoclinous stalked antheridia making a broad apical contact zone with the oogonia	1 per oogonium, arising from the oogonial stalk
Oospore	Globose, aplerotic, one per oogonium, 12.2–23.3 μm	Single, rarely 2, globose, plerotic or aplerotic, 12.5–22 μm in diameter	Aplerotic, usually single, occasionally 2 oospores per oogonium, globose, 17–23 μm in diameter.	Spherical, elongated and irregular, 1 to 3 per oogonia, plerotic, rarely aplerotic, 12–22 μm in diameter	Plerotic rarely aplerotic, mostly spherical, single, 12–21 μm in diameter
Wall thickness of oospore	0.6–2.4 μm	1.2–1.8 μm	1.5–2.5 μm	1–2 μm	1 μm

a Ahadi et al. (2024).

b Ali-Shtayeh and Dick (1985).

c Uzuhashi et al. (2010).

d Paul and Bala (2008).

e Hendrix (1974).

## 4 Pathogenicity assay

**Figure 4:**
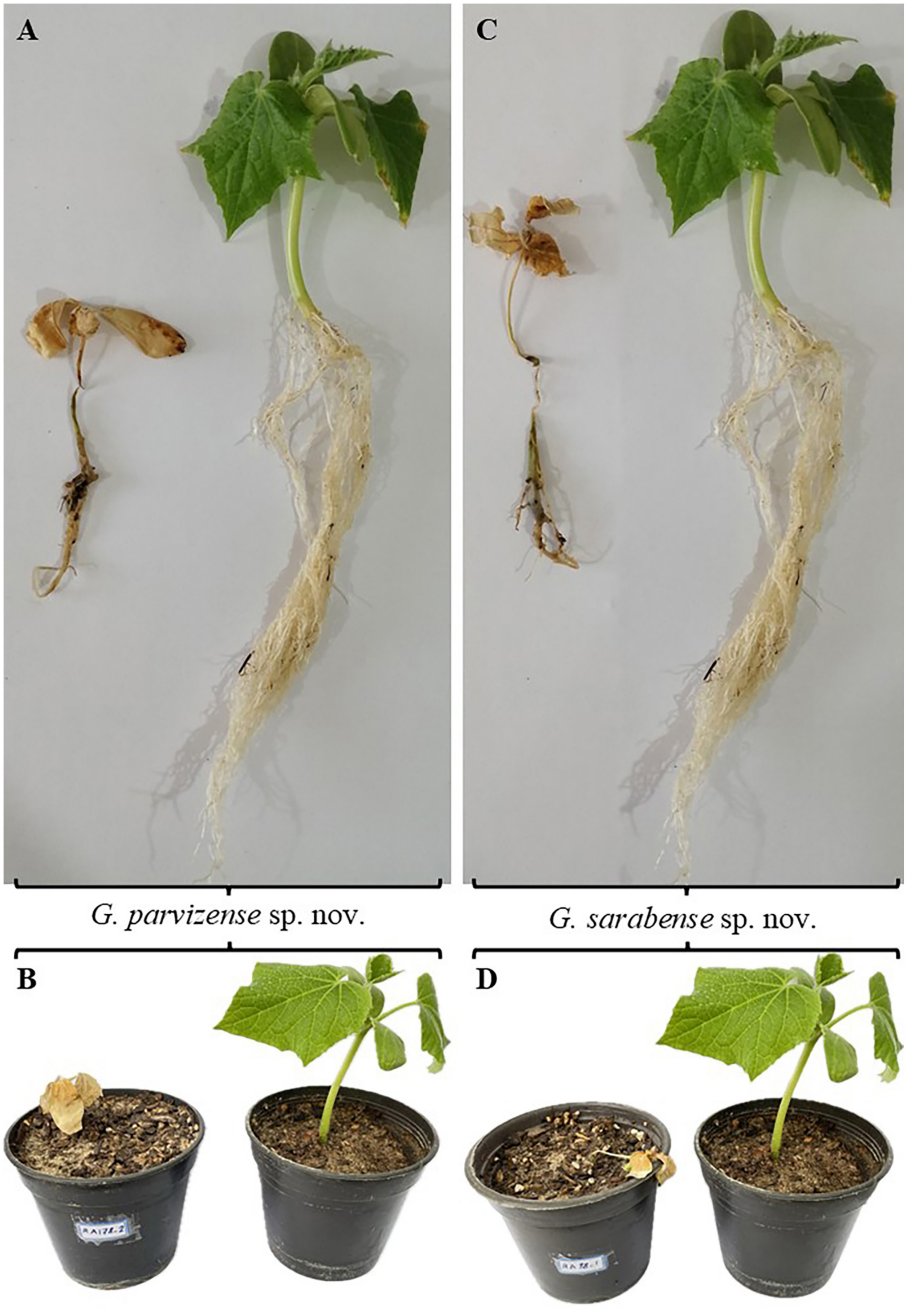
Pathogenicity test results. **(A, B)** Plants inoculated with *Globisporangium parvizense* sp. nov. (IRAN 5217C) on the left; corresponding negative control plants on the right. **(C, D)** Plants inoculated with *G. sarabense* sp. nov. (IRAN 5173C) on the left; corresponding negative control plants on the right.

Pathogenicity experiments confirmed the pathogenicity of all examined isolates, including *G. parvizense* sp. nov. (IRAN 5217C) and *G. sarabense* sp. nov. (IRAN 5173C), resulting in crown and root rot and subsequent seedling death (Figure 4). All inoculated seedlings developed characteristic water-soaked lesions at the crown, leading to crown and root rot and eventually plant collapse within 2 weeks.

Seedlings emerged normally 3 days post-sowing (dps). However, by 5 dps, initial symptoms—water-soaked lesions on the crown accompanied by incipient wilting—became apparent. These symptoms were intensified by 7 dps, with lesion coalescence, stem browning, and tissue decay extending into the root system. Control plants (negative control) remained symptomless throughout the experiment and exhibited significantly greater root and stem development compared to the inoculated treatments. Microscopical examination of infected tissues revealed the presence of sporangia and oospores in all three tested isolates. Furthermore, successful re-isolation of the original isolates from infected plants (100%) and their subsequent identification through detailed microscopy and morphometry fulfilled Koch's postulates, unequivocally confirming their pathogenicity.

## 5 Discussion

This study was part of a broader research project aimed at investigating the diversity of *Pythium* s.l. populations from Iranian aquatic environments. The primary focus of this survey was to study the diversity of *Globisporangium* species in non-agricultural environments in Iran. We described two new species of *Globisporangium*, namely *G. parvizense* sp. nov. and *G. sarabense* sp. nov., using a multi-gene phylogenetic framework that combined nuclear rDNA ITS sequences with partial sequences of cytochrome c oxidase subunits I and II (*cox1* and *cox2*), supported by morphological assessments. The multi-gene phylogenetic approach enabled a comparative evaluation of the discriminatory robustness of these three genetic markers—ITS, *cox1*, and *cox2*—based on their nucleotide-level variation among the newly described species.

The newly described species can be clearly differentiated from previously described species in *Globisporangium* based on their morphological characteristics. Morphologically, *G. parvizense* differs from its close relatives in several ways: it lacks zoospores (unlike *G. schmitthenneri* and *G. hypogynum*), has a discharge tube (unlike *G. acrogynum*), possesses one to two antheridia per oogonium (unlike *G. schmitthenneri* and *G. acrogynum*), and forms two oospores per oogonium (unlike *G. acrogynum* and *G. hypogynum*). Similarly, *G. sarabense* can be distinguished by its uniquely shaped sporangia, absence of zoospores (unlike *G. lucens* and *G. debaryanum*), lack of oogonium chains (unlike *G. tabrizense* and *G. lucens*), and presence of a single antheridium and oospore per oogonium (in contrast to *G. tabrizense, G. lucens*, and *G. viniferum*).

For *G. sarabense*, the ITS region exhibited 21 and 15 nucleotide differences compared to *G. lucens* and *G. tabrizense*, respectively, making it the most informative marker for distinguishing these species. In contrast, *cox1* showed 18 and 17 differences, while *cox2* showed 10 and 12, respectively. Thus, while all three loci contributed to species delimitation, ITS provided the highest resolution in this case. Conversely, in *G. parvizense*, ITS showed only two to three nucleotide differences compared to *G. hypogynum, G. schmitthenneri*, and *G. acrogynum*, indicating limited discriminatory power. *Cox1* showed four differences, while *cox2* showed seven, making *cox2* the most informative marker in this lineage.

These findings underscore that the effectiveness of genetic markers can vary across lineages. While ITS proved most effective for *G. sarabense*, the mitochondrial genes (*cox1* and *cox2*) offered superior resolution for *G. parvizense*. This highlights (I) the limitations of relying solely on ITS—commonly used as a DNA barcode for oomycetes—which may lack sufficient resolution for some taxa, and (II) the importance of a multi-locus phylogenetic approach for robust species delimitation, providing a deeper understanding of the genetic diversity and evolutionary relationships within *Pythium* s.l.

The isolation of two novel *Globisporangium* species from irrigation systems and their positive pathogenicity toward cucumber plants might suggest that such organisms are not merely transient inhabitants but may actively exploit freshwater systems as a means of dispersal, raising concerns about their potential role in introducing plant diseases into agricultural settings. This is particularly important in Iran as it is one of the top cucumber producers globally and grows it widely near rivers and other natural water bodies (Nikolaou et al., 2021).

Moreover, the confirmed pathogenicity of these species on cucumber raises important ecological questions about the ability of aquatic-associated oomycetes to transition from water-associated habitats to terrestrial plant hosts. Although cucumber is not an aquatic plant, its constant exposure to irrigation water, flooding, and high soil moisture creates an ecological bridge that allows aquatic-origin oomycetes to colonize terrestrial crops. This highlights the importance of future research into the environmental adaptability of these pathogens and their potential infection under natural field conditions (Redekar et al., 2020).

The findings of this study highlight the urgent need for systematic monitoring and risk assessment of oomycetes in irrigation systems. If left unchecked, these pathogens could pose a serious threat to food security and sustainable agriculture. Future work should aim to monitor oomycete populations in agricultural water, identify high-risk species, and develop targeted disease management strategies to prevent future outbreaks.

## Data Availability

The datasets presented in this study can be found in online repositories. The names of the repository/repositories and accession number(s) can be found in the article/supplementary material.
